# Psychometric properties and confirmatory factor analysis of the CASP-19, a measure of quality of life in early old age: the HAPIEE study

**DOI:** 10.1080/13607863.2014.938605

**Published:** 2014-07-25

**Authors:** Gyu Ri Kim, Gopalakrishnan Netuveli, David Blane, Anne Peasey, Sofia Malyutina, Galina Simonova, Ruzena Kubinova, Andrzej Pajak, Simone Croezen, Martin Bobak, Hynek Pikhart

**Affiliations:** ^a^ Department of Epidemiology and Public Health, University College London, London, UK; ^b^ Institute for Health and Human Development, University of East London, London, UK; ^c^ Institute of Internal and Preventive Medicine, Siberian Branch under the Russian Academy of Medical Sciences, Novosibirsk, Russia; ^d^ Novosibirsk State Medical University, Novosibirsk, Russia; ^e^ Environmental Health Monitoring System, National Institute of Public Health, Prague, Czech Republic; ^f^ Department of Epidemiology and Population Sciences, Faculty of Health Sciences, Institute of Public Health, Jagiellonian University Medical College, Krakow, Poland; ^g^ Department of Public Health, Erasmus University Medical Center, Rotterdam, The Netherlands

**Keywords:** quality of life, CASP-19, well-being, psychometrics, old age

## Abstract

**Objectives:** The aim was to assess the reliability and validity of the quality of life (QoL) instrument CASP-19, and three shorter versions of CASP-12 in large population sample of older adults from the HAPIEE (Health, Alcohol, and Psychosocial factors In Eastern Europe) study.

**Methods:** From the Czech Republic, Russia, and Poland, 13,210 HAPIEE participants aged 50 or older completed the retirement questionnaire including CASP-19 at baseline. Three shorter 12-item versions were also derived from original 19-item instrument. Psychometric validation used confirmatory factor analysis, Cronbach's alpha, Pearson's correlation, and construct validity.

**Results:** The second-order four-factor model of CASP-19 did not provide a good fit to the data. Two-factor CASP-12v.3 including residual covariances for negative items to account for the method effect of negative items had the best fit to the data in all countries (CFI = 0.98, TLI = 0.97, RMSEA = 0.05, and WRMR = 1.65 in the Czech Republic; 0.96, 0.94, 0.07, and 2.70 in Poland; and 0.93, 0.90, 0.08, and 3.04 in Russia). Goodness-of-fit indices for the two-factor structure were substantially better than second-order models.

**Conclusions:** This large population-based study is the first validation study of CASP scale in Central and Eastern Europe (CEE), which includes a general population sample in Russia, Poland, and the Czech Republic. The results of this study have demonstrated that the CASP-12v.3 is a valid and reliable tool for assessing QoL among adults aged 50 years or older. This version of CASP is recommended for use in future studies investigating QoL in the CEE populations.

## Background

With declining mortality rates and increased longevity, there has been a substantial increase in the proportion of adults reaching older age. In Europe, the number of older people aged 65 and over has risen significantly over the past two decades. The percentage of Europeans aged 65 or older is projected to rise from 17.4% at present to 27% in 2058 (Eurostat, 2012). With such an increase, governments worldwide are concerned with how to promote healthy ageing and assist older people to maintain their independence and active participation in society, in effect, to enhance quality of life (QoL) at older ages.

There is no general consensus regarding how QoL should be defined or measured. However, most researchers consider QoL to be a multi-dimensional concept encompassing various concepts including life satisfaction, and covers physical, emotional, mental health, as well as social and behavioural components of well-being (Bergner, 1989; Janse et al., 2004). The WHO Quality of Life Group defines QoL as: ‘An individuals’ perception of their position in life in the context of the culture and value systems in which they live and in relation to their goals, expectations, standards, and concerns’ (Kuyken et al., 1995). QoL can be assessed by both objective and subjective measures: an individual's financial situation and general expectations from life as well as other factors such as education, housing, social support, and health. Within the gerontological literature, aging has traditionally been conceived in terms of physiological, mental, and social decline and deterioration in health. As such, QoL at older ages has been predominantly measured by health-related quality of life measures, for example, the Short Form-36 (SF-36), to assess the effects of poor health on mental and physical functioning (Lima et al., 2011; Walters, Munro, & Brazier, 2001; Ware & Sherbourne, 1992). As older people live longer and healthier , it is no longer appropriate to reduce QoL in older people to their experience of physical and mental health alone, and there is a need for a measure of QoL that explores positive experiences of ageing.

In the present study, we focus on the 19-item Control, Autonomy, Self-realisation, Pleasure scale (CASP-19). CASP-19 is a theoretically based measure of broader QoL, which was specially designed to measure QoL in the ‘Third Age’ (Blane, Higgs, Hyde, & Wiggins, 2004; Higgs, Hyde, Wiggins, & Blane, 2003; Wiggins, Higgs, Hyde, & Blane, 2004). Third Age has been characterised as a period of life after retirement, in which one is free from many social roles, and able to explore areas of personal fulfilment. CASP-19 is underpinned by social theories: specifically, Maslow, Doyal, and Gough's theory on human need (Doyal & Gough, 1991; Maslow, 1968); Laslett's theory of the Third Age (Laslett, 1989); Giddens on reflexivity (Giddens, 1991); and Gilleard and Higgs on cultures of ageing (Gilleard & Higgs, 2000). QoL is defined in terms of satisfaction of human needs in four life domains, namely control, autonomy, self-realisation, and pleasure. CASP-19 aims to capture positive aspects of life at older ages, whilst being independent of factors, including financial circumstance and health that may influence it. The control domain represents people's ability to actively control their environments, whilst autonomy is defined as the freedom from unwanted interference of others. Self-realisation and pleasure domains capture the active and self-reflexive aspects of living that bring reward and happiness to people in later life (Higgs et al., 2003).

CASP-19 has been extensively used in various samples and settings. CASP items have been administered in large population-based samples such as the English Longitudinal Study of Ageing (ELSA) waves 1–5 (Marmot, Banks, Blundell, Lessof, & Nazroo, 2003), the 2004 U.S. Health and Retirement Study (Clarke, Fisher, House, Smith, & Weir, 2007); and the British Household Panel Study (BHPS) (Taylor, Brice, Buck, & Prentice-Lane, 2001). Two shorter versions are also available: CASP-12v.1 was developed for the use in the Survey of Health, Ageing and Retirement in Europe (Borsch-Supan et al., 2013; Borsch-Supan et al., 2005) and CASP-12v.2 (Wiggins, Netuveli, Hyde, Higgs, & Blane, 2008).

There have been a number studies which have validated the factor structure of CASP-19 (Bowling & Stenner, 2011; Sexton, King-Kallimanis, Conroy, & Hickey, 2013; Sim, Bartlam, & Bernard, 2011; Vanhoutte, 2012; Vanhoutte, 2014; Wiggins et al., 2008; Wu et al., 2013). Most of the studies on the psychometric properties of CASP-19 have been conducted in West European countries, primarily in the United Kingdom (Sim et al., 2011; Wiggins et al., 2008) and Ireland (Sexton et al., 2013), but also in Taiwan (Wu et al., 2013). In earlier studies of CASP, a four-factor solution was suggested for CASP-19 and two- or three-factor structure for 12-item CASP scales. In a UK study, Wiggins et al. (2008) has shown that a four-factor measurement model of CASP-19 has a good fit to the data in the BHPS and ELSA samples, using the confirmatory factor analysis (CFA) approach. Also, the shortened three-factor CASP-12v.2 proved slightly superior to the original 19-item scale. These findings have been confirmed by Sim et al. (2011); the authors assessed the psychometric properties of CASP-19 and CASP-12v.2 on a sample of 120 British adults living in the retirement community. More recently, Sexton et al. (2013) undertook a detailed psychometric assessment of CASP-19 using The Irish Longitudinal Study of Ageing (TILDA) (Table 1). Their findings did not support the validity of the established measurement models. The control and autonomy, self-realisation, and pleasure factors were not sufficiently distinctive either empirically or conceptually. Instead, they recommended the use of a revised 12-item scale with either a single-factor or two-factor model (CASP-12v.3) when assessing overall QoL (Sexton et al., 2013). Also, studies have found a method effect in the CASP scale and allowing error correlations between negatively worded items led to significant improvement in model fit (Sexton et al., 2013; Vanhoutte, 2014).

To date, the psychometric properties of the proposed single- or two-factor CASP-12v.3 have not yet been further investigated in other studies. The aim of this study is to establish the reliability and validity of the original 19-item and 12-item CASP scales in the sample of older adults living in Central and Eastern Europe (CEE). In addition, factor structure of the newly suggested CASP-12v.3 instrument will be further investigated using CFA.

In the past 20 years, the countries of CEE have experienced a remarkable social and economic transition from closed, totalitarian, and centrally planned economies towards open, democratic, and market-based economies. Such transition has had devastating impact on health. In the early 1990s, many countries in CEE experienced a dramatic increase in mortality rates. The largest increase was concentrated in the Former Soviet Union (FSU). In Russia, for example, male life expectancy at birth decreased from 63.8 to 57.7 years and 74.4 to 71.2 years for women between 1990 and 1994 (Notzon et al., 1998). This natural experiment offers a unique opportunity to study the health outcomes and well-being in these countries as they undergo significant socio-economic restructuring. Validation of CASP instrument in CEE will be useful for determining its potential for the use in future research and for comparing the QoL of older adults with other international studies which have incorporated the CASP.

## Methods

### Study population

The study subjects come from wave 1 of the HAPIEE (Health, Alcohol, and Psychosocial factors In Eastern Europe) project. The sampling procedure and design of HAPIEE is described in detail elsewhere (Peasey et al., 2006), and is briefly summarised below. HAPIEE comprises random population samples of men and women aged 45–70 years in Novosibirsk (Russia), Krakow (Poland), and seven Czech towns – Jihlava, Havirov, Hradec Kralove, Karvina, Kromeriz, Liberec, and Usti nad Labem. The data for wave 1 of the study were collected in 2002–2005. At baseline, a total of 28,945 individuals have been recruited (response rate 59%). In this study, we analysed data from participants who were administered the retirement questionnaire including the QoL (CASP-19) questions. Participants also completed an extensive questionnaire on their medical history, health status, lifestyle, diet, and socio-economic and psychosocial factors, and underwent a short clinical examination for measurement of anthropometric parameters. All questions were translated from English into each language and back translated into English to check for accuracy.

### Measurements

#### Quality of life

QoL was assessed in all participants who were retired using CASP-19. CASP-19 is a self-completed 19-item questionnaire originally developed and validated in a representative sample of 263 adults aged 65–75 years from the UK Boyd Orr Study (Gunnell, Frankel, Nanchahal, Braddon, & Smith, 1996). Each scale item was rated on 4-point Likert scale, with responses ranging from 0 (never), 1 (not very often), 2 (sometimes), and 3 (often). The scale includes both positively and negatively worded items (see Appendix 1). All negatively worded questions were reverse coded so that all item responses are in the same direction. The total scores ranged from 0 to 57 for CASP-19. Higher scores indicate better QoL. The original CASP scale is composed of 19 items, but revised forms of 12 items (Borsch-Supan et al., 2005; Sexton et al., 2013; Wiggins et al., 2008) have been proposed for use. In the original study that tested the qualities of the CASP scale, a four-dimensional structure was proposed for the 19-item scale (Hyde, Wiggins, Higgs, & Blane, 2003), and both single- or two-factor (Sexton et al., 2013) and three-factor structures have been proposed for the different 12-item versions of CASP (Borsch-Supan et al., 2013; Wiggins et al., 2008). CASP-12v.1 (Borsch-Supan et al., 2013; Borsch-Supan et al., 2005) and CASP-12v.2 (Wiggins et al., 2008) were derived from CASP-19 by removing items which correlated most weakly with other items in their dimensions. CASP-12v.1 consists of 12 items: C1, C2, C4, A5, A6, A9, P10, P11, P14, S15, S18, and S19. CASP-12v.2 (Wiggins et al., 2008) was composed of items C1, C2, C4, A5, A7, A9, P10, P11, P12, S15, S18, and S19 (See Appendix 1). For the proposed three-factor structure, items in the control and autonomy domains were merged together to form a single component, while items related to pleasure and self-realisation represent separate dimensions. In recent studies, Sexton et al. (2013) and Vanhoutte (2014) have indicated that there is a method effect in the CASP scale, and a revised CASP instrument with residual covariances for the negatively worded items (method factor) should be included. Sexton et al. (2013) have indicated that a revised 12-item, one- or two-factor model comprising control/autonomy and self-realisation/pleasure domains, with residual covariances for negatively worded items, had excellent fit to the data. CASP-12v.3 scale included items C1, C2, C3, C4, A7, A8, A9, P10, P11, P13, S17, and S18 (Appendix 1).

### Socio-demographic variables

In addition to CASP items, the following variables were used for the purpose of obtaining the descriptive statistics: the marital status was categorised into four groups (married or cohabiting, single (never married), divorced, and widowed), the educational level was categorised into four groups (primary or less, vocational, secondary, and university), health status was divided into three groups (very good and good, average, and poor and very poor), level of material deprivation was assessed by three questions about the frequency of difficulties in (1) paying bills, (2) buying food, and (3) clothes necessary for themselves and/or his family. The answers were recorded on a 5-point scale (coded from 0 to 4); the total deprivation score was calculated as the sum of the three questions, and categorised into three groups: low (0), medium (1–6), and high (7–12). Self-reported economic activity was classified into the following categories: working pensioners and non-working pensioners.

Participants in Poland and the Czech Republic completed the questionnaire during a nurse visit to their home (85% of them subsequently attended an examination in a clinic); all Russian participants completed the questionnaire during a visit to a clinic.

### Statistical analyses

#### Sample description and initial steps of the analysis

Data were analysed in STATA version 12 for descriptive analyses and Mplus version 6.11 for CFAs (Muthén & Muthén, 1998–2010). The analytical strategy was as follows. First, descriptive statistics, chi-squared tests, and ana-lysis of variance (ANOVA) test were employed to describe the data. Second, frequency distributions were examined to evaluate the normality of scale items. Missing data and floor and ceiling effects (percentages of participants indicating minimum and maximum scores) of the CASP-19 were investigated in order to verify the validity and reliability of scale content (Ware & Gandek, 1998). Such effects were considered to be present if more than 15% of the sample reported the lowest or highest score (McHorney & Tarlov, 1995; Terwee et al., 2007). If floor or ceiling effects are present, it is likely that extreme items are missing in the lower or upper end of the scale. In such cases, as a result, participants with the lowest or highest possible scores cannot be distinguished from each other, and the reliability of the questionnaire is reduced. Missing item responses up to 10% have been considered as acceptable (af Sandeberg, Johansson, Hagell, & Wettergren, 2010).

Second, internal consistency reliability of CASP-19 was determined using Cronbach's alpha (α). It evaluates the extent to which items within a scale are inter-correlated with one another and measures the same concept. Cronbach's alpha typically ranges from 0 to 1. Internal consistency reliability is suggested to be acceptable when Cronbach's alpha ≥ 0.70 (DeVellis, 1991). Item-total correlations were calculated to examine the dimensionality of the scale items. Items within each dimension should represent the same latent variable and correlate more strongly with own domain than others. This is considered satisfactory if item-total correlations are ≥0.40 (Ware & Gandek, 1998). Construct validity was further examined by analysing the correlation between CASP-19 dimensions with other previously validated measures (Cohen, 2005). Spearman's correlation coefficients were used and were interpreted as follows: >0.90: excellent relationship, 0.71–0.90: good, 0.51–0.70: fair, 0.31–0.50: weak, and ≤0.30: none.

Previous QoL studies have sought to find evidence of construct validity by correlating with other established measures such as the SF-36, self-rated health status, and satisfaction with life scales (Bowling, 2009; Sim et al., 2011). Two measures that have been incorporated in the HAPIEE questionnaire were used for this purpose: physical functioning and self-rated health status. Physical functioning was measured by the 10 questions on activities of daily living from the SF-36 questionnaire (Mchorney, Ware, & Raczek, 1993; Ware & Sherbourne, 1992). Respondents were asked to rate their health over the last 12 months (1 = good/very good; 2 = average; 3 = poor/very poor). Higher self-rated health scores indicate poorer heath, and a negative correlation with the CASP-19 would be hypothesised. Conversely, physical functioning was rated on a 0–100 scale, with a higher score indicating better physical functioning. CASP-19 should correlate positively with physical functioning. Correlation coefficients between 0.1 and 0.3 are considered low, between 0.3 and 0.5 moderate, and over 0.5 high.

### Confirmatory factor analysis (CFA)

**Figure 1.**
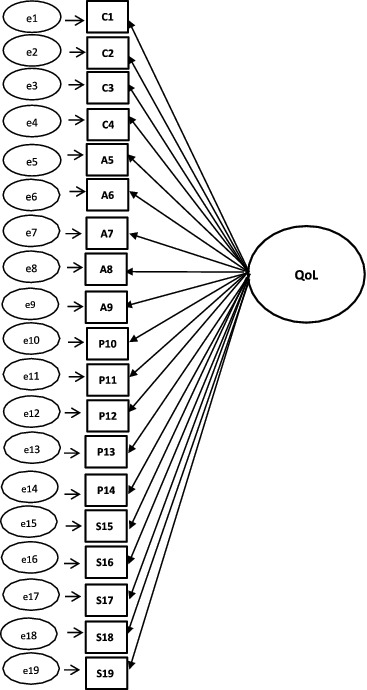
Single-factor model for CASP-19.

**Figure 2.**
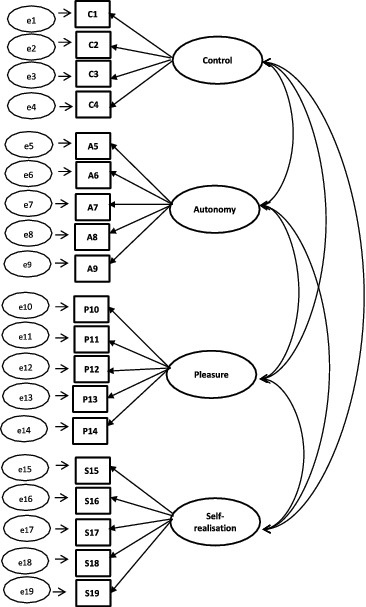
First-order model for CASP-19.

**Figure 3.**
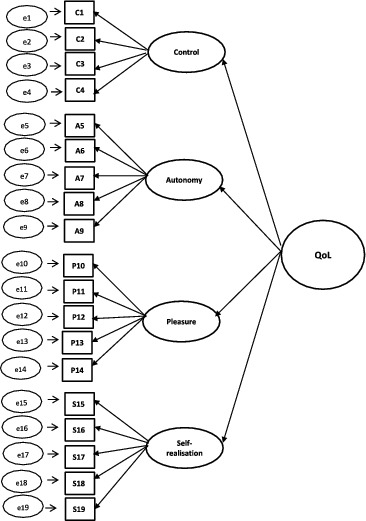
Second-order model for CASP-19.

**Figure 4.**
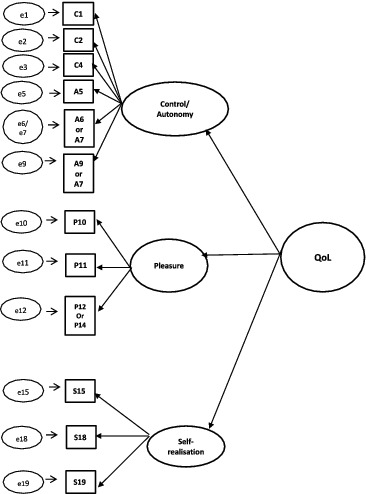
Second-order model for 12-item CASP.

For the CFA of CASP-19, CASP-12v.1, and CASP-12v.2, three competing models were tested: (1) single-factor model, (2) first-order model, and (3) second-order model. A schematic representation of the models can found in Figures 1–4.

**Figure 5.**
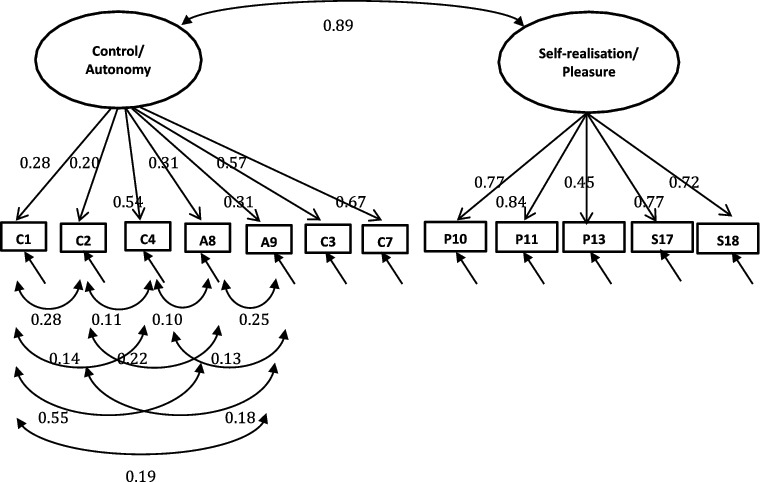
CASP-12v.3 two-factor measurement model with standardised loadings for the Czech Republic.

A single-factor model where all 19 or 12 items load directly onto unobserved variable called QoL was tested (Figure 1), followed by a first-order model in which the four domains were included (Figure 2). In the second-order measurement model, the CASP domains are allowed to be dependent upon a single underlying factor, QoL (Figures 3 and 4). The second-order model is applicable when (1) the lower order factors are highly correlated with each other, and (2) there is a higher order factor which is hypothesised to account for the relations among the lower order factors. A second-order factor solution with four domains was proposed for the 19-item scale, and a similar factor structure based on three domains was proposed for CASP-12v.1 and CASP-12v2. In addition, we examined the single- and two-factor structures of CASP-12v.3 as proposed by Sexton et al. (2013) (see Figure 5). The two-factor model is composed of control/autonomy and self-realisation/pleasure factors, and this includes residual covariances for negative items, to take account of method effect that arises from the direction of wording in the scale items (Marsh, 1996).

CFA was computed using the weighted least square estimator with a mean- and variance-adjusted chi-squared method to handle ordered categorical items as dependent variables in Mplus. Missing data across CASP-19 were handled using full information maximum likelihood estimation. This method computes parameter estimates on the basis of all available data, including the incomplete cases. The procedure works under the assumption that the data are missing at random.

### Assessing the degree of model fit

To evaluate overall model fit, three goodness-of-fit indices were calculated. These indices include comparative fit index (CFI), Tucker–Lewis index (TLI), root mean square error of approximation (RMSEA), and weighted root mean square residual (WRMR). According to Hu and Bentler (1999), a CFI value of greater than 0.90 can be expected for a psychometrically acceptable fit to the data. RMSEA is another quantitative index which describes how well the model fits the observed data. As a rule of thumb, the value of RMSEA <0.05 indicates good fit, values between 0.05 and 0.08 suggest acceptable model fit, and values >0.10 suggest poor model fit. For the CFI and TLI, values above 0.90 can be expected for a reasonably good fitting model. For the WRMR, values <1.0 have been suggested as indicative of adequate model fit (Hancock & Mueller, 2006).

### Ethical approval and consent

The study received ethical approval from the local ethical committees in each participating country and at University College London, and all participants gave written consent.

## Results

### Sample characteristics

Of all the 14,059 retirees (approximately 50%; 5906 males and 8153 females), we restricted inclusion in the study to those aged 50–70 years who answered at least one of the CASP-19 items. There were 449 retirees who did not give any responses to the CASP-19 questionnaire and were, therefore, excluded from the analyses. Also, there were participants younger than 50, who answered the module for retired people. These respondents are most likely to be retired for health reasons. There were also few respondents aged 70 or older but their number was low and they would not well represent this age group. Consequently, 400 subjects who were outside the 50–70 years range were excluded. Thus, the analytical sample consisted of 13,210 individuals (Czech Republic: *n* = 3782; Russia: *n* = 3802; Poland: *n* = 5626).

The baseline descriptive characteristics of the 13,210 individuals with valid data are shown in Table 2. The mean age of participants in all countries ranged between 62 and 64 years for men and 61 and 63 years for women. A large majority of participants were married and had completed vocational or secondary level of education.

**Table 1.** Model-fit statistics for a second-order measurement model for CASP-19, using CFA.

Study	Sample	*N*	CFI	TLI	RMSEA
Wiggins et al. (2008)	BHPS, wave 11	6471	0.76	0.91	0.09
Wiggins et al. (2008)	ELSA, wave 1	9300	0.76	0.91	0.13
Sim et al. (2011)	UK retirement community	120	0.86	0.90	0.12
Sexton et al. (2013)	The Irish Longitudinal Study of Ageing (TILDA)	6823	0.85	0.83	0.10
Wu et al. (2013)	Community- dwelling Taiwanese residents	699	0.73	0.69	0.11

*N* = sample size; CFI = comparative fit index, values >0.90 indicate good fit; TLI = Tucker–Lewis index, values >0.90 indicate good fit; RMSEA = root mean square error of approximation, values <0.05 indicate good fit.

**Table 2.** Descriptive characteristics of the study population at baseline.

	Men (*N* = 5527)			Women (*N* = 7683)		
	Czech towns (*N* = 1579)	Russia (*N* = 1364)	Poland (*N* = 2584)	Czech towns (*N* = 2203)	Russia (*N* = 2438)	Poland (*N* = 3042)
Age (Mean, SD)	64.4 (4.2)	64.0 (4.5)	62.6 (5.1)	63.1 (4.3)	63.1 (4.6)	61.8 (5.2)
						
CASP-19 score (Mean, SD)	35.5 (9.9)	34.5 (9.3)	38.0 (9.3)	34.3 (10.2)	33.1 (8.5)	36.8 (8.9)
CASP-12v.1 score (Mean, SD)	22.0 (6.6)	20.8(6.1)	24.4 (6.2)	21.1 (6.9)	19.8 (5.7)	23.7 (6.1)
CASP-12v.2 score (Mean, SD)	22.8 (6.7)	21.4 (6.4)	25.2(6.3)	22.0 (6.8)	20.6 (5.8)	24.8 (6.1)
CASP-12v.3 score	22.0 (6.5)	21.3 (6.3)	24.2 (6.0)	21.1 (6.7)	20.4 (5.7)	23.7 (5.8)
						
Marital status (%)						
Married or cohabiting	85.1	85.1	85.0	65.1	54.4	62.0
Single, divorced/separated	9.3	7.6	8.9	14.2	16.3	13.9
Widowed	5.5	7.3	5.8	20.4	29.3	24.0.
Missing	0.1	0.0	0.3	0.3	0.0	0.1
						
Education level (%)						
Primary	8.0	21.0	13.9	23.5	15.9	19.0
Vocational	46.4	20.9	31.5	33.4	27.1	15.8
Secondary	31.1	33.5	34.0	35.6	37.7	45.3
University	13.7	24.6	20.5	7.0	19.3	19.8
Missing	0.8	0.0	0.1	0.5	0.0	0.1
						
Health status						
Very good and good	27.4	8.3	25.5	30.0	3.0	20.4
Average	54.8	60.3	54.5	54.9	62.0	58.7
Poor and very poor	17.2	31.4	19.9	14.4	35.0	20.7
Missing	0.6	0.0	0.1	0.7	0.0	0.2
						
Material deprivation						
Low (0)	53.3	23.7	51.6	44.4	15.2	41.6
Medium (1–6)	39.9	45.0	37.6	47.4	50.0	43.2
High (7–12)	4.9	31.3	9.4	6.0	34.8	14.2
Missing	1.9	0.0	1.4	2.2	0.0	1.0
						
Economic activity						
Pensioner, still employed	12.2	–	13.5	7.7	–	8.7
Pensioner, not employed	87.0	100.0	86.5	91.4	100.0	91.2
Missing	0.8			0.9	0.0	0.1

There were differences in socio-demographic characteristics between the samples. Notably, Russians reported higher levels of poor/very poor health than Czechs (women 35.0% vs. 14.2%), while also presenting lower rates of very good/good health (women 3.0% vs. 30.3%; *P* < 0.001). Also, the Russians reported higher levels of self-reported material deprivation. The proportion of participants with university education was higher in Russia, and in all countries it was higher in men than in women. Compared to Czech and Polish women, there was a large proportion of widows and lower proportion of married or cohabiting women in Russia. Four times as many Russian women (29.3%) were widowed, compared to men (7.3%). In regards to economic status, the Russian sample consisted only of non-working pensioners, while proportion of Czech and Poles still working ranged between 7.7% and 13.5%, respectively.

There were significant differences in mean values of CASP between countries (*p* < 0.001). Men scored significantly higher on the CASP-19 than women in all countries. Polish men and women reported the highest CASP-19 scores (mean score: 38.0 (95% CI = 37.7, 38.4) for men and 36.8 (95% CI = 36.5, 37.1) for women). The lowest CASP-19 scores were reported by Russian men (mean score: 34.5 (95% CI = 32.8, 33.4)). Distribution of CASP-19 was skewed in all countries (skewness: −0.96, −0.09, and −0.28 for the Czech Republic, Russia, and Poland, respectively). In the Czech sample, the median CASP-19 score was 36. Median scores for each CASP-19 sub-scale were as follows: control = 6 (inter-quartile range [IQR] = 4–8), autonomy = 9 (IQR = 7–11), self-realisation = 8 (IQR = 6–10), and pleasure = 13 (IQR = 11–15). Among Russian participants, median scores for each CASP-19 sub-scale were as follows: control = 6 (IQR = 4–8), autonomy = 8 (IQR = 7–10), self-realisation = 7 (IQR = 4–10), and pleasure = 13 (IQR = 10–15). In Poland, median scores were control = 7 (IQR = 5–9), autonomy = 8 (IQR = 6–10), self-realisation = 10 (IQR = 8–12), and pleasure = 14 (IQR = 12–15).

The distributions of responses to each of the items in the CASP are shown in Appendix 2. Most of the participants completed all 19 items (*n* = 12,692; 93.3%). Missing data were relatively small, with between 0.5% and 6.7% respondents not providing a response to an item. A marked ceiling effect was found in the pleasure domain, with the highest ceiling effect of 67.3% (Czech Republic), 70.4% (Russia), and 76.2% (Poland).

### CASP reliability

Table 3 shows the Cronbach's alpha coefficients for the four CASP scales. CASP-19 scale presented acceptable to good internal consistency coefficients. Cronbach's alpha of CASP-19 total score was 0.84 (Czech Republic), 0.83 (Russia), and 0.86 (Poland). Nearly all CASP subscales had high internal consistency. Self-realisation domain had respectable reliability, with coefficient alpha ranging from 0.73 to 0.75. The pleasure subscale was found to be highly reliable (α = 0.78, α = 0.74, and α = 0.75 for Czech Republic, Russia, and Poland, respectively). However, autonomy domains had particularly low reliability coefficients, which suggests unacceptable reliability (DeVellis, 1991). When the control and autonomy domains were combined together to form the 12-item scale, alpha coefficient for the domain rose to 0.56, 0.68, and 0.63 (CASP-12v.1), and 0.58, 0.69 and 0.68 (CASP-12v.2) for the Czech Republic, Russia, and Poland, respectively. The CASP-12v.3 score and its subscales had high reliability coefficients varying between 0.76 and 0.80 for the three HAPIEE samples. With the exception of control/autonomy domain in the Czech Republic (α = 0.64), all subscale coefficients were close to or above 0.70.

**Table 3.** Cronbach's alpha coefficient of internal consistency reliability.

	Czech Republic	Russia	Poland
CASP-19 total score	0.84	0.83	0.86
Control	0.47	0.63	0.62
Autonomy	0.53	0.58	0.57
Pleasure	0.78	0.72	0.78
Self-realisation	0.73	0.74	0.75
CASP-12v.1 total score	0.78	0.74	0.79
Control + autonomy	0.56	0.68	0.63
Pleasure	0.74	0.57	0.69
Self-realisation	0.72	0.70	0.73
CASP-12v.2 total score	0.80	0.77	0.82
Control + autonomy	0.58	0.69	0.68
Pleasure	0.78	0.66	0.79
Self-realisation	0.77	0.70	0.73
CASP-12v.3 total score	0.76	0.76	0.80
Control + autonomy	0.64	0.71	0.72
Self-realisation + pleasure	0.75	0.69	0.72

Moreover, each item of CASP-19 was correlated with total scores for both its own domain and the other three domains. All 19 items had high correlations with their respective domains (See Appendix 3).

### Correlations between CASP-19 and physical functioning, self-rated health, CESD-20: evidence for construct validity

The associations of CASP-19 dimensions with physical functioning scales, self-rated health, and CESD-20 are shown in Table 4. Physical functioning (SF-10) and self-rated health scores were moderately correlated with total CASP-19 score in each country. These findings indicated that as the level of physical functioning increases, the QoL increases. Conversely, the level the QoL decreases with increasing levels of depressive symptoms and poor self-rated health. All correlations were significant at *p* < 0.001.

**Table 4.** Correlation coefficients of the dimensions of CASP-19 with physical functioning (SF-10), self-rated health, and CESD-20 depression scale.

	Czech Republic			Russia			Poland		
	SF-10	Self-rated health	CESD-20	SF-10	Self-rated health	CESD-20	SF-10	Self-rated health	CESD-20
CASP-19 total scale	0.40	−0.41	−0.49	0.40	−0.37	−0.40	0.41	−0.37	−0.57
Control	0.32	−0.31	−0.39	0.34	−0.30	−0.34	0.34	−0.28	−0.47
Autonomy	0.35	−0.34	−0.34	0.34	−0.32	−0.21	0.38	−0.33	−0.39
Self-realisation	0.35	−0.38	−0.40	0.32	−0.27	−0.41	0.36	−0.33	−0.49
Pleasure	0.24	−0.27	−0.41	0.18	−0.20	−0.20	0.22	−0.24	−0.50

Notes: All correlations were significant at *P* < 0.001.

Spearman's correlation coefficients are interpreted as follows: >0.90 = excellent relationship; 0.90, 0.90–0.71 = good; 0.70–0.51 = fair; 0.51-0.31 = weak, ≤0.30 = none.

### Confirmatory factor analysis (CFA)

Table 5 presents the goodness-of-fit indices for the three measurement models in each country.

The four-factor solutions for CASP-19 had relatively poor model fit, as illustrated by the goodness-of-fit indices. RMSEA values were all above or equal to 0.10; CFI and TLI values were below 0.90. Although the three-factor second-order model suggested the best fit of all the three models, the fit indices indicted only an acceptable model in the Czech and Polish samples (Czech Republic: CFI = 0.96, TLI = 0.94, RMSEA = 0.08; Poland: CFI = 0.96, TLI = 0.95, RMSEA = 0.07 for CASP-12v.2) and a marginal model fit for Russia (CASP-12v.2: CFI = 0.86, TLI = 0.82, RMSEA = 0.16).

In addition to the established measurement models, a model composed of control/autonomy and self-realisation/pleasure factors which included error correlations between negative items were tested. Two-factor model of CASP-12v.3 was good fit to the data in the Czech Republic (CFI = 0.98, TLI = 0.97, RMSEA = 0.05, WRMR = 1.65) and satisfactory model fit in Poland and Russia (Poland: CFI = 0.96, TLI = 0.94, RMSEA = 0.07 WRMR = 2.70; Russia: CFI = 0.93, TLI = 0.90, RMSEA = 0.08, WRMR = 3.04). Goodness-of-fit indices for the two-factor structure were substantially better than the second-order models (Table 6). Similarly, the single-factor measurement model provided a good fit to the data, suggesting that either single-factor or two-factor models fit the data equally well.

**Table 5:** Goodness-of-fit indices for the three measurement models for CASP-19, CASP-12v.1, and CASP-12v.2 in the HAPIEE wave 1.

	Czech Republic			Russia			Poland		
Measures	CFI	TLI	RMSEA	CFI	TLI	RMSEA	CFI	TLI	RMSEA
CASP-19									
Single-factor model	0.86	0.85	0.10	0.73	0.69	0.15	0.85	0.83	0.11
First-order model : four-factor	0.90	0.88	0.09	0.83	0.80	0.12	0.89	0.87	0.10
Second-order model: four-factor	0.88	0.86	0.10	0.81	0.78	0.12	0.87	0.85	0.10
CASP-12v.1									
Single-factor model	0.92	0.90	0.09	0.69	0.62	0.17	0.89	0.86	0.11
First-order model: three-factor	0.96	0.95	0.07	0.89	0.84	0.11	0.96	0.94	0.08
Second-order model: three-factor	0.95	0.93	0.08	0.83	0.78	0.13	0.94	0.92	0.08
CASP-12v.2									
Single-factor model	0.93	0.91	0.09	0.72	0.66	0.17	0.90	0.88	0.11
First-order model: three-factor	0.88	0.84	0.12	0.88	0.84	0.12	0.96	0.95	0.07
Second-order model: three-factor	0.96	0.94	0.08	0.86	0.82	0.13	0.96	0.95	0.07

Note: CFI = comparative fit index, values >0.90 indicate good fit; TLI = Tucker–Lewis index, values >0.90 indicate good fit; RMSEA = root mean square error of approximation, values <0.05 indicate good fit.

**Table 6.** Goodness-of-fit indices for the two-factor model with correlated errors for negative items in the HAPIEE wave 1.

		Czech Republic				Russia				Poland			
Measures		CFI	TLI	RMSEA	WRMR	CFI	TLI	RMSEA	WRMR	CFI	TLI	RMSEA	WRMR
CASP-19	1-factor	0.94	0.93	0.07	2.89	0.80	0.79	0.13	5.69	0.90	0.88	0.09	4.62
CASP-19	2-factor	0.95	0.94	0.07	2.68	0.83	0.79	0.12	5.30	0.92	0.89	0.09	4.28
CASP-12v.1	1-factor	0.95	0.93	0.07	2.69	0.85	0.72	0.13	5.05	0.93	0.91	0.09	3.87
CASP-12v.1	2-factor	0.96	0.94	0.07	2.43	0.87	0.82	0.11	4.30	0.95	0.93	0.08	3.31
CASP-12v.2	1-factor	0.95	0.94	0.08	2.72	0.84	0.78	0.14	5.27	0.94	0.92	0.08	3.74
CASP-12v.2	2-factor	0.96	0.94	0.08	2.55	0.86	0.81	0.13	4.76	0.95	0.93	0.09	3.52
CASP-12v.3	1-factor	0.98	0.96	0.05	1.79	0.91	0.87	0.09	3.50	0.95	0.93	0.07	3.02
CASP-12v.3	2-factor	0.98	0.97	0.05	1.65	0.93	0.90	0.08	3.04	0.96	0.94	0.07	2.70

Notes: CFI = comparative fit index, values >0.90 indicate good fit; TLI = Tucker–Lewis index, values >0.90 indicate good fit; RMSEA = root mean square error of approximation, values <0.05 indicate good fit.

CASP-12v.1: C1, C2, C4, A5, A6, A9, P10, P11, P14, S15, S18, and S19.

CASP-12v.2: C1, C2, C4, A5, A7, A9, P10, P11, P12, S15, S18, and S19.

CASP-12v.3: C1, C2, C3, C4, A7, A8, A9, P10, P11, P13, S17, and S18.

**Figure 6.**
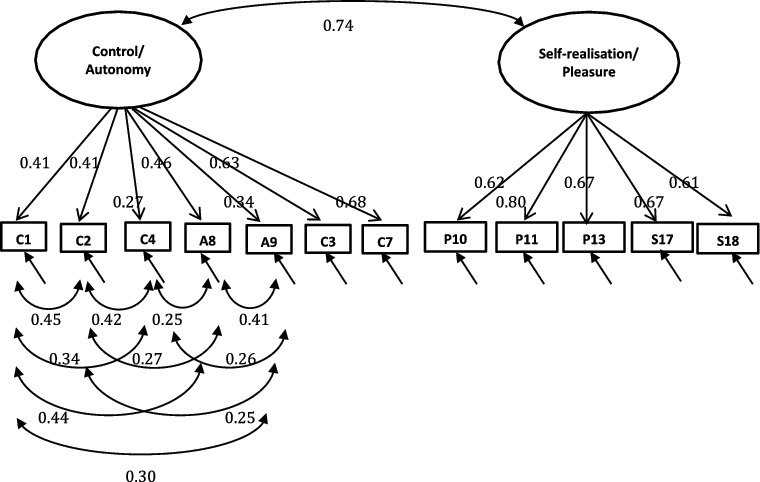
CASP-12v.3 two-factor measurement model with standardised loadings for Russia.

**Figure 7.**
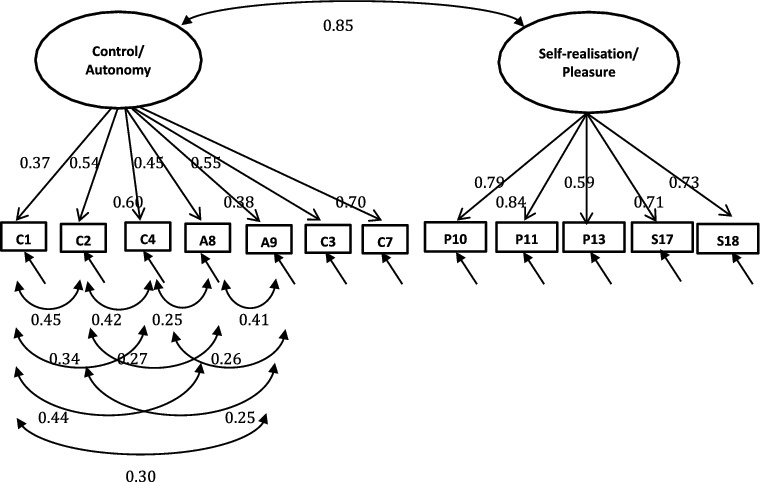
CASP-12v.3 two-factor measurement model with standardised loadings for Poland.

For two-factor measurement models of CASP-12v.3, all item-factor loadings were significant (*p* < 0.001). Items on the self-realisation/pleasure exhibited strong factor loadings (>0.40) for all the three samples. Four items were below the 0.4 level in the Czech sample (items C1, C2, A8, and A9), whereas two items did not reach the recommended 0.4 threshold in the Russian and Polish samples (items C4, A9, and items C1 and A9 in Russian and Poland, respectively) (see Figures 5–7). Item C1 – ‘my age prevents me from doing the things I would like to do’– and item A9 – ‘shortage of money stops me from doing the things I want to do’– exhibited lower factor loadings than other items among all samples (Czech Republic: 0.28, 0.31; Russia: 0.41, 0.34; Poland: 0.37 0.38 for C1). Moreover, the correlation between the control/autonomy and self-realisation/pleasure factors was significant and very high (Czech Republic: *r* = 0.89; Russia: *r* = 0.74; Poland: r = 0.85). This indicates that there may be only one factor underlying the 12 items of CASP.

## Discussion

The purpose of this research was to evaluate the psychometric properties of the CASP scales in a sample of older adults aged 50 or over living in CEE. This large population-based study in CEE is the first validation study of CASP scales in this population group, which includes a general population sample in Russia, Poland, and the Czech Republic.

Given the mortality crisis, higher levels of cardiovascular diseases, and the high prevalence of unhealthy behaviours in Russia (Bobak, McKee, Rose, & Marmot, 1999; Bobak et al., 2002; Bobak et al., 2004; Netuveli, Pikhart, Bobak, & Blane, 2012), we expected mean CASP-19 scores to be lower in Russia than in the Czech Republic or Poland. In both genders, the mean score of CASP-19 was the lowest in Russia (men: 34.5, 95% CI = 34.0–35.0; women: 33.1, 95% CI = 32.8–33.4), while Polish men (Mean = 38.0, 95% CI = 37.7–38.4) and women (Mean = 36.8, 95% CI = 36.5–37.1) reported the highest CASP-19 scores. These scores are significantly lower than the mean score reported for the ELSA (Mean = 42.5, 95% CI = 42.3–42.7) (Netuveli, Wiggins, Hildon, Montgomery, & Blane, 2006) and TILDA (Mean = 43.8, 95% CI = 43.6–44.1) (Layte, Sexton, & Savva, 2013).

We found a skewed distribution for almost all items of CASP-19. Consequently, the ceiling effect was high which indicated that the full range of the scale was not captured. High ceiling effect can affect the responsiveness of the CASP questionnaire (Terwee et al., 2007), which reduces the scale's ability to discriminate amongst degrees of better QoL. However, the reliability analysis revealed a satisfactory internal consistency estimate. Our estimated alpha coefficients are in line with those reported by Wiggins et al. (2008). Cronbach's alphas are greater than 0.70 for all subscales with the exception of autonomy subscale.

Consistent with the existing literature, there was little evidence of good fit for the second-order model using CASP-19; RMSEA values were all above or equal to 0.10; CFI and TLI values were below, which indicated unsatisfactory model fit. Our results of CFA suggest that the ‘second-order model’ has adequate fit to the data for the Czech and Polish samples. CFI and TLI values are greater than 0.9 which is above Hu & Bentler's (1999) cut-off criteria for fit indices. For Russia, the ‘second-order’ model had a marginal model fit to the data. These results suggested that the CASP scales could be revised further to achieve better model fit.

It is difficult to compare our results to other CEE/FSU data, due to lack of similar local studies. However, our results of CFA are in agreement with the evidence from UK studies. For CASP-12v.2, the goodness-of-fit indices of the latter two models are of a similar magnitude as that found by Wiggins et al. (2008) (BHPS wave 11: CFI = 0.91; TLI = 0.96; RMSEA = 0.07). Also, our CFI and TLI values for CASP-12v.1 are comparable to Vanhoutte's work on CASP using ELSA wave 1 participants (CFI = 0.94, TLI = 0.93, RMSEA = 0.09) (Vanhoutte, 2012). In regards to CASP-12v.3, our findings are in accordance with the study by Sexton et al. (2013) (two-factor model: CFI = 0.99, TLI = 0.99, RMSEA = 0.03, WRMR = 1.76).

The Russian data were somewhat less well fit by the proposed measurement models than Czech and Polish data. This discrepancy in results across HAPIEE populations may be attributed to issues surrounding translation artefact, cultural relevance of certain CASP items, and variation in the interpretation of items across respondents of different cultures (Ramirez, Ford, Stewart, & Teresi, 2005). Certain CASP questions may have slightly different connotations in one language than another.

Although the countries of CEE/FSU share some socio-economic and political characteristics, the analysed group of countries is still little heterogeneous in terms of their geography, natural resources, democratic structure, and developmental trajectories. Historically, governments in these countries followed different overall socio-economic transformation policies after the collapse of communism in 1989: shock therapy in Russia and more social–liberal approach in the Czech Republic and Poland. There is also divergence in the range of health indicators, such as life expectancy or cardiovascular disease (CVD) trends, socio-economic trajectories, and alcohol consumption patterns in the region. For example, in 2011, the life expectancies at age 45 years in Russia, Poland, the Czech Republic, and the European Union were 28.6, 33.1, 34.0, and 36.4, respectively (WHO, 2011). In general, CEE countries have better health outcomes than FSU countries. Due to this heterogeneity, the operationalisation of CASP and some items are likely to have different cultural meaning or value for those from CEE and FSU.

The study has a number of limitations. First, the CASP-19 is a self-completed questionnaire. A methodological problem commonly associated with the use of self-report measures, which may have been present in our study, is the inability to determine the extent to which responses accurately reflect the respondents’ experiences due to inaccurate recall; respondents for various reasons may under or overestimate their QoL. Second, since the Russian data only comprised non-working subjects, the working pensioners are excluded from the analysis. Consequently, respondents included in the study may not be representative of the whole population and the generalisation of our results may be limited. Thus, future studies with a more heterogeneous group of participants are needed to examine the psychometric properties in more detail. Finally, the data used in this study had been collected in 2002–2005, and the results reflect conditions in these countries at the time of data collection which might be little different from the conditions in these countries now. We, however, believe that 15 years between the start of political and social changes and data collection have been long enough to make these societies more stable and the results are still applicable to current societies in the region.

## Conclusion

Despite the above-mentioned limitations, this is one of the first, and the largest study so far on the levels and psychometric properties of CASP in CEE. In conclusion, CASP-12v.3 is a valid and reliable tool for assessing QoL among older adults aged 50 years or older. This version of CASP is recommended for the use in future studies investigating QoL in the CEE populations.

## Appendix 1. Item wording for CASP-19

**Table:**

(Often = 3, Not Often = 2, Sometimes = 1, and Never = 0)		
Control	C1	My age prevents me from doing the things I would like to do.
	C2	I feel that what happens to me is out of my control.
	C3	I feel free to plan for the future.
	C4	I feel left out of things.
		
Autonomy	A5	I can do the things that I want to do.
	A6	Family responsibilities prevent from doing what I want to do.
	A7	I feel that I can please myself what I can do.
	A8	My health stops me from doing the things I want to do.
	A9	Shortage of money stops me from doing the things I want to do.
		
Pleasure	P10	I look forward to each day.
	P11	I feel that my life has meaning.
	P12	I enjoy the things that I do.
	P13	I enjoy being in the company of others.
	P14	On balance I look back on my life with a sense of happiness.
		
Self-realisation	S15	I feel full of energy these days.
	S16	I choose to do things I have never done before.
	S17	I feel satisfied with the way my life has turned out.
	S18	I feel that life is full of opportunities.
	S19	I feel that the future looks good for me.

**Table:**

Item wordings for CASP-12v.1, CASP-12v.2, and CASP-12v.3			v.1	v.2	v.3
Control/autonomy	C1	My age prevents me from doing the things I would like to do	x	x	x
	C2	I feel that what happens to me is out of my control	x	x	x
	C3	I feel free to plan for the future			x
	C4	I feel left out of things	x	x	x
	A5	I can do the things that I want to do	x	x	
	A6	Family responsibilities prevent from doing what I want to do	x		
	A7	I feel that I can please myself what I can do		x	x
	A8	My health stops me from doing the things I want to do			x
	A9	Shortage of money stops me from doing the things I want to do	x	x	x
					
Pleasure^1^	P10	I look forward to each day	x	x	x
	P11	I feel that my life has meaning	x	x	x
	P12	I enjoy the things that I do		x	
	P13	I enjoy being in the company of others			x
	P14	On balance I look back on my life with a sense of happiness	x		
					
Self-realisation^1^	S15	I feel full of energy these days	x	x	
	S17	I feel satisfied with the way my life has turned out			x
	S18	I feel that life is full of opportunities	x	x	x
	S19	I feel that the future looks good for me	x	x	

Note: Negative-worded items (C1, C2, A4, A6, A8, and A9) were reverse coded.

^1^Self-realisation and pleasure would form one factor in CASP-12v.3.

## Appendix 2. Item response proportions and % missing values for the CASP-19 scale

**Table:**

	Czech Republic					Russia					Poland				
Response	0	1	2	3	Missing	0	1	2	3	Missing	0	1	2	3	Missing
Item	(%)	(%)	(%)												
C1	16.2	45.4	20.5	12.5	5.4	30.7	37.8	13.7	17.8	0	20.8	32.3	21.0	25.2	0.7
C2	23.5	41.7	17.1	12.2	5.5	13.2	47.7	20.1	19.0	0	8.9	24.2	20.2	45.6	1.1
C3	16.7	24.0	34.7	18.4	6.2	16.5	19.4	29.5	34.6	0	25.5	38.7	25.4	9.5	0.9
C4	3.7	20.0	23.4	46.3	6.6	17.8	44.2	19.0	19.0	0	3.6	13.9	15.9	65.1	1.5
A5	5.3	11.6	32.4	45.6	5.1	4.5	14.0	33.1	48.4	0	14.2	27.7	29.9	27.3	0.9
A6	6.1	26.8	29.1	30.7	7.3	8.9	28.7	21.0	41.4	0	13.8	26.1	21.1	37.8	1.2
A7	1.9	6.4	34.9	51.5	5.3	1.9	3.8	27.6	66.7	0	2.3	8.6	34.9	53.3	0.9
A8	22.7	37.9	22.0	12.9	4.5	42.5	39.7	8.9	8.9	0	36.6	33.8	14.1	14.7	0.8
A9	24.7	37.5	19.5	13.1	5.2	54.2	32.4	8.1	5.3	0	47.7	33.2	10.8	7.6	0.7
P10	0.9	4.6	20.1	71.1	3.3	6.3	11.2	19.8	62.7	0	1.2	4.0	18.1	76.2	0.5
P11	1.3	5.8	28.4	60.1	4.4	5.2	16.0	24.9	53.9	0	1.4	4.3	19.3	74.2	0.8
P12	0.5	3.4	25.4	67.3	3.4	1.8	4.8	28.8	64.6	0	1.2	4.6	23.3	70.1	0.8
P13	0.7	3.8	31.2	60.3	4.0	1.2	4.0	24.4	70.4	0	0.6	4.0	24.9	70.0	0.5
P14	1.6	9.2	39.6	45.4	4.2	4.7	8.3	26.0	61.0	0	4.6	13.6	33.7	47.0	1.1
S15	4.6	23.0	45.5	21.3	5.6	22.0	36.6	26.9	14.5	0	6.0	23.0	41.3	28.8	0.9
S16	21.3	34.2	27.5	10.4	6.6	34.7	26.2	31.5	7.6	0	26.5	32.5	28.7	11.2	1.1
S17	5.0	12.1	40.8	37.5	4.6	14.9	19.5	30.4	35.2	0	6.4	10.9	28.6	53.0	1.1
S18	12.9	36.1	32.0	12.3	6.7	23.7	21.2	24.3	30.8	0	2.8	11.9	29.3	55.1	0.9
S19	9.5	26.0	41.4	17.0	6.1	37.9	20.8	24.8	16.5	0	8.0	21.0	36.6	33.3	1.1

Note: Each item was rated on a 4-point scale from 0 (never), 1 (not often), 2 (sometimes), and 3 (often).

## Appendix 3. Spearman's correlations between CASP items and domains for the Czech Republic, Russia, and Poland.

**Table:**

	Czech Republic				Russia				Poland			
Item	Control	Autonomy	Pleasure	Self-realisation	Control	Autonomy	Pleasure	Self-realisation	Control	Autonomy	Pleasure	Self-realisation
1	**0**.**647**	0.417	0.160	0.239	**0**.**746**	0.426	0.173	0.256	**0**.**731**	0.439	0.251	0.258
2	**0**.**619**	0.235	0.142	0.186	**0**.**741**	0.426	0.170	0.169	**0**.**769**	0.379	0.327	0.308
3	**0**.**621**	0.359	0.328	0.435	**0**.**611**	0.332	0.301	0.399	**0**.**549**	0.406	0.316	0.463
4	**0**.**618**	0.379	0.354	0.295	**0**.**652**	0.355	0.116	0.112	**0**.**638**	0.336	0.350	0.275
5	0.353	**0**.**639**	0.343	0.389	0.423	**0**.**712**	0.394	0.317	0.447	**0**.**681**	0.326	0.374
6	0.204	**0**.**535**	0.116	0.084	0.182	**0**.**589**	0.160	-0.104	0.181	**0**.**519**	0.071	-0.003
7	0.324	**0**.**557**	0.467	0.427	0.292	**0**.**602**	0.436	0.121	0.379	**0**.**520**	0.498	0.397
8	0.439	**0**.**606**	0.167	0.293	0.475	**0**.**615**	0.219	0.331	0.478	**0**.**680**	0.251	0.364
9	0.308	**0**.**612**	0.179	0.252	0.389	**0**.**549**	0.106	0.192	0.292	**0**.**589**	0.218	0.288
10	0.274	0.269	**0**.**657**	0.422	0.117	0.202	**0**.**698**	0.312	0.304	0.283	**0**.**649**	0.382
11	0.349	0.303	**0**.**765**	0.519	0.215	0.285	**0**.**766**	0.468	0.362	0.282	**0**.**696**	0.445
12	0.285	0.330	**0**.**694**	0.451	0.291	0.461	**0**.**727**	0.275	0.371	0.383	**0**.**708**	0.456
13	0.161	0.164	**0**.**583**	0.273	0.218	0.378	**0**.**665**	0.189	0.247	0.242	**0**.**614**	0.351
14	0.287	0.306	**0**.**741**	0.501	0.160	0.251	**0**.**659**	0.248	0.338	0.324	**0**.**783**	0.572
15	0.397	0.404	0.488	**0**.**707**	0.429	0.329	0.324	**0**.**692**	0.457	0.450	0.499	**0**.**742**
16	0.030	0.034	0.109	**0**.**461**	0.146	0.054	0.217	**0**.**573**	0.123	0.142	0.181	**0**.**585**
17	0.355	0.396	0.588	**0**.**699**	0.224	0.299	0.486	**0**.**656**	0.346	0.322	0.557	**0**.**692**
18	0.355	0.352	0.487	**0**.**783**	0.228	0.179	0.342	**0**.**756**	0.377	0.313	0.523	**0**.**680**
19	0.422	0.412	0.523	**0**.**779**	0.192	0.089	0.329	**0**.**761**	0.387	0.345	0.544	**0**.**767**

Note: Items in bold form each respective domain.

## References

[cit0001] af Sandeberg M., Johansson E.M., Hagell P., Wettergren L. (2010). Psychometric properties of the DISABKIDS chronic generic module (DCGM-37) when used in children undergoing treatment for cancer. *Health and Quality of Life Outcomes*.

[cit0002] Bergner M. (1989). Quality of life, health-status, and clinical research. *Medical Care*.

[cit0003] Blane D., Higgs P., Hyde M., Wiggins R.D. (2004). Life course influences on quality of life in early old age. *Social Science & Medicine*.

[cit0004] Bobak M., McKee M., Rose R., Marmot M. (1999). Alcohol consumption in a national sample of the Russian population. *Addiction*.

[cit0005] Bobak M., Murphy M., Pikhart H., Martikainen P., Rose R., Marmot M. (2002). Mortality patterns in the Russian Federation: Indirect technique using widowhood data. *Bulletin of the World Health Organization*.

[cit0006] Bobak M., Room R., Pikhart H., Kubinova R., Malyutina S., Pajak A., Marmot M. (2004). Contribution of drinking patterns to differences in rates of alcohol related problems between three urban populations. *Journal of Epidemiology and Community Health*.

[cit0007] Borsch-Supan A., Brandt M., Hunkler C., Kneip T., Korbmacher J., Malter F., Zuber S. (2013). Data resource profile: The survey of health, ageing and retirement in Europe (SHARE). *International Journal of Epidemiology*.

[cit0008] Borsch-Supan A., Brugiavini A., Jurges H., Mackenbach J., Siegrist J., Weber G. (2005). *Health ageing and retirement in Europe: First results from the survey of health, ageing and retirement in Europe*.

[cit0009] Bowling A. (2009). The psychometric properties of the older people's quality of life questionnaire, compared with the CASP-19 and the WHOQOL-OLD. *Current Gerontology and Geriatric Research*.

[cit0010] Bowling A., Stenner P. (2011). Which measure of quality of life performs best in older age? A comparison of the OPQOL, CASP-19 and WHOQOL-OLD. *Journal of Epidemiology and Community Health*.

[cit0011] Clarke P., Fisher G., House J., Smith J., Weir D. (2007). *Guide to content of the HRS psychosocial leave-behind participant lifestyle questionnaires: 2004 & 2006*.

[cit0012] Cohen R.J. (2005). *Exercises in psychological testing and assessment*.

[cit0013] DeVellis R.F. (1991). *Scale development : Theory and applications*.

[cit0014] Doyal L., Gough I. (1991). *A theory of human need*.

[cit0015] Eurostat (2012). *Active ageing and solidarity between generations: A statistical portrait of the European Union 2012: A statistical portrait of the European Union 2012*.

[cit0016] Giddens A. (1991). *The consequences of modernity*.

[cit0017] Gilleard C.J., Higgs P. (2000). *Cultures of ageing: Self, citizen, and the body*.

[cit0018] Gunnell D.J., Frankel S., Nanchahal K., Braddon F.E., Smith G.D. (1996). Life course exposure and later disease: A follow-up study based on a survey of family diet and health in pre-war Britain (1937–1939). *Public Health*.

[cit0019] Hancock G.R., Mueller R.O. (2006). *Structural equation modeling: A second course*.

[cit0020] Higgs P., Hyde M., Wiggins R., Blane D. (2003). Researching quality of life in early old age: The importance of the sociological dimension. *Social Policy & Administration*.

[cit0021a] Hu L., Bentler P.M. (1999). Cutoff criteria for fit indexes in covariance structure analysis: Conventional criteria versus new alternatives. *Structural Equation Modeling: A Multidisciplinary Journal*.

[cit0021] Hyde M., Wiggins R.D., Higgs P., Blane D.B. (2003). A measure of quality of life in early old age: The theory, development and properties of a needs satisfaction model (CASP-19). *Aging Mental Health*.

[cit0022] Janse A.J., Gemke R.J.B.J., Uiterwaal C.S.P.M., van der Tweel I., Kimpen J.L.L., Sinnema G. (2004). Quality of life: Patients and doctors don't always agree: A meta-analysis. *Journal of Clinical Epidemiology*.

[cit0023] Kuyken W., Orley J., Power M., Herrman H., Schofield H., Murphy B., Vandam F. (1995). The World Health Organization Quality of Life assessment (WHOQOL) – position paper from the World Health Organization. *Social Science & Medicine*.

[cit0024] Laslett P. (1989). *A fresh map of life: The emergence of the third age*.

[cit0025] Layte R., Sexton E., Savva G. (2013). Quality of life in older age: Evidence from an Irish cohort study. *Journal of American Geriatrics Society*.

[cit0026] Lima M.G., Barros M.B.D., Cesar C.L.G., Goldbaum M., Carandina L., Alves M.C.G.P. (2011). Health-related behavior and quality of life among the elderly: A population-based study. *Revista de Saude Publica*.

[cit0027] Marmot M., Banks J., Blundell R., Lessof C., Nazroo J. (2003). *The health, wealth and lifestyles of older populations in England. The 2002 English longitudinal study of aging*.

[cit0028] Marsh H.W. (1996). Positive and negative global self-esteem: A substantively meaningful distinction or artifactors. *Journal of Personality and Social Psychology*.

[cit0029] Maslow A.H. (1968). *Toward a psychology of being*.

[cit0030] McHorney C.A., Tarlov A.R. (1995). Individual-patient monitoring in clinical practice: Are available health status surveys adequate. *Quality of Life Research*.

[cit0031] Mchorney C.A., Ware J.E., Raczek A.E. (1993). The Mos 36-item short-form health survey (Sf-36) .2. Psychometric and clinical-tests of validity in measuring physical and mental-health constructs. *Medical Care*.

[cit0032] Muthén L.K., Muthén B.O. (1998–2010). *Mplus user's guide*.

[cit0033] Netuveli G., Pikhart H., Bobak M., Blane D. (2012). Generic quality of life predicts all-cause mortality in the short term: Evidence from British household panel survey. *Journal of Epidemiology and Community Health*.

[cit0034] Netuveli G., Wiggins R.D., Hildon Z., Montgomery S.M., Blane D. (2006). Quality of life at older ages: Evidence from the English longitudinal study of aging (wave 1). *Journal of Epidemiology and Community Health*.

[cit0035] Notzon F.C., Komarov Y.M., Ermakov S.P., Sempos C.T., Marks J.S., Sempos E.V. (1998). Causes of declining life expectancy in Russia. *JAMA*.

[cit0036] Peasey A., Bobak M., Kubinova R., Malyutina S., Pajak A., Tamosiunas A., Marmot M. (2006). Determinants of cardiovascular disease and other non-communicable diseases in Central and Eastern Europe: Rationale and design of the HAPIEE study. *BMC Public Health*.

[cit0037] Ramirez M., Ford M.E., Stewart A.L., Teresi J.A. (2005). Measurement issues in health disparities research. *Health Services Research*.

[cit0038] Sexton E., King-Kallimanis B.L., Conroy R.M., Hickey A. (2013). Psychometric evaluation of the CASP-19 quality of life scale in an older Irish cohort. *Quality of Life Research*.

[cit0039] Sim J., Bartlam B., Bernard M. (2011). The CASP-19 as a measure of quality of life in old age: Evaluation of its use in a retirement community. *Quality of Life Research*.

[cit0040] Taylor M., Brice J., Buck N., Prentice-Lane E. (2001). *British household panel survey-user manual-volume a: Introduction, technical report and appendices*.

[cit0041] Terwee C.B., Bot S.D., de Boer M.R., van der Windt D.A., Knol D.L., Dekker J., de Vet H.C. (2007). Quality criteria were proposed for measurement properties of health status questionnaires. *Journal of Clinical Epidemiology*.

[cit0042] Vanhoutte B. (2012). *Measuring subjective well-being in later life: Review*.

[cit0043] Vanhoutte B. (2014). The multidimensional structure of subjective well-being in later life. *Journal of Population Ageing*.

[cit0044] Walters S.J., Munro J.F., Brazier J.E. (2001). Using the SF-36 with older adults: A cross-sectional community-based survey. *Age Ageing*.

[cit0045] Ware J.E., Gandek B. (1998). Methods for testing data quality, scaling assumptions, and reliability: The IQOLA Project approach. International Quality of Life Assessment. *Journal of Clinical Epidemiology*.

[cit0046] Ware J.E., Sherbourne C.D. (1992). The Mos 36-Item Short-Form Health Survey (Sf-36) .1. Conceptual-framework and item selection. *Medical Care*.

[cit0047] World Health Organization (2011). *European health for all database (HFA-DB)*.

[cit0048] Wiggins R.D., Higgs P.F.D., Hyde M., Blane D.B. (2004). Quality of life in the third age: Key predictors of the CASP-19 measure. *Ageing & Society*.

[cit0049] Wiggins R.D., Netuveli G., Hyde M., Higgs P., Blane D. (2008). The evaluation of a self-enumerated scale of quality of life (CASP-19) in the context of research on ageing: A combination of exploratory and confirmatory approaches. *Social Indicators Research*.

[cit0050] Wu T.Y., Chie W.C., Kuo K.L., Wong W.K., Liu J.P., Chiu S.T., Blane D. (2013). Quality of life (QOL) among community dwelling older people in Taiwan measured by the CASP-19, an index to capture QOL in old age. *Archives of Gerontology and Geriatrics*.

